# Clinical and prognostic significance of circulating levels of angiopoietin-1 and angiopoietin-2 in hepatocellular carcinoma

**DOI:** 10.18632/oncotarget.26507

**Published:** 2018-12-28

**Authors:** Roberto Carmagnani Pestana, Manal M. Hassan, Reham Abdel-Wahab, Yehia I. Abugabal, Lauren M. Girard, Donghui Li, Ping Chang, Kanwal Raghav, Jeff Morris, Robert A. Wolff, Asif Rashid, Hesham M. Amin, Ahmed Kaseb

**Affiliations:** ^1^ Department of Cancer Medicine, The University of Texas MD Anderson Cancer Center, Houston, TX, USA; ^2^ Department of Epidemiology, The University of Texas MD Anderson Cancer Center, Houston, TX, USA; ^3^ Department of Gastrointestinal Medical Oncology, The University of Texas MD Anderson Cancer Center, Houston, TX, USA; ^4^ Department of Biostatistics, The University of Texas MD Anderson Cancer Center, Houston, TX, USA; ^5^ Department of Pathology, The University of Texas MD Anderson Cancer Center, Houston, TX, USA; ^6^ Department of Hemopathology, The University of Texas MD Anderson Cancer Center, Houston, TX, USA; ^7^ Department of Clinical Oncology, Assiut University Hospitals, Faculty of Medicine, Assiut, Egypt

**Keywords:** angiogenesis, angiopoietin-1, angiopoietin-2, cirrhosis, hepatocellular carcinoma

## Abstract

Angiopoietin-1 (Ang-1) and angiopoietin-2 (Ang-2) play critical roles in angiogenesis in hepatocellular carcinoma (HCC). In addition, recent data suggest that Ang-1/Ang-2 are involved in regulating the immune response. The aim of our study was to explore the clinical prognostic significance of plasma Ang-1 and Ang-2 in HCC. We prospectively enrolled and collected data and blood samples from 767 HCC patients treated at MD Anderson Cancer Center between 2001 and 2014. Controls consisted of cirrhotic patients (*n* = 75) and healthy volunteers (*n* = 200). The cutoff value was the median level of each angiogenic factor. Overall survival (OS) was estimated by Kaplan–Meier curves and compared by the log-rank test. Higher plasma Ang-2 was significantly associated with advanced clinicopathologic features of advanced HCC and lower OS. Median OS was 61.8 months (95% confidence interval [CI], 45.1–78.5 months) for low Ang-2 compared with 28.5 months (95% CI, 24.8–32.1 months) for high Ang-2 (*p* < 0.001). In contrast, higher Ang-1 was associated with longer OS. Median OS was 37.2 months (95% CI, 31.0–43.4 months) for high Ang-1 compared with 26.2 months (95% CI, 22.2–30.3 months) for those with low Ang-1 (p = 0.043). In conclusion, our findings indicate that plasma Ang-1 and Ang-2 levels are potential diagnostic and prognostic biomarkers in HCC.

## INTRODUCTION

Hepatocellular carcinoma (HCC), the most common primary liver cancer and the second leading cause of cancer-related death in the world, is responsible for approximately 750,000 deaths per year [1]. Multiple factors are involved in the high mortality rates of HCC, including ineffective screening and lack of access to treatment in some regions [2–4]. In addition, given the asymptomatic nature in its early stages, most HCC cases are detected at an advanced stage, leading to incurable disease states in patients with low hepatic functional reserve [4–6]. The development of HCC is closely related to the presence of chronic liver disease, and the most prevalent underlying etiologic factors are viral hepatitis B and C, exposure to aflatoxins, alcohol use, and nonalcoholic steatohepatitis caused by metabolic syndrome [7, 8]. Surgical resection, liver transplantation, and radiofrequency ablation are the only curative therapeutic modalities for localized HCC, and treatment options for patients with locally advanced or metastatic disease are limited [9, 10].

Sorafenib and lenvatinib, orally available multikinase inhibitors, have demonstrated modest overall survival (OS) benefit and are currently the only systemic therapies approved for first-line treatment of advanced HCC [11, 12]. More recently, immunotherapy was incorporated into the therapeutic arsenal for HCC, as nivolumab was approved in the second-line setting [13]. However, validated predictive and prognostic biomarkers to advance personalized oncologic management, and predict therapeutic outcomes and survival are lacking for this highly aggressive cancer [14].

Notably, HCC development, progression, and metastasis are strongly associated with angiogenesis pathway [11]. Accordingly, antiangiogenic approaches are part of the standard of care in HCC treatment; lenvatinib and sorafenib, for example, target the vascular endothelial growth factor receptor (VEGFR), among other kinases [12]. An imbalance between proangiogenic and antiangiogenic factors is believed to drive angiogenesis in HCC [15, 16]. Angiopoietin-1 (Ang-1) and angiopoietin-2 (Ang-2) were first described as ligands for Tie2, a transmembrane receptor tyrosine kinase that regulates survival, proliferation, and migration of endothelial cells [17–19]. In physiologic conditions, homeostatic balance is maintained between Ang-1 and Ang-2 activity [17–19]. Ang-1 is primarily expressed in periendothelial support cells of quiescent blood vessels [11]. Binding of Ang-1 to Tie2 promotes structural integrity of the vascular networks [17–21]. In contrast, Ang-2 is expressed during inflammation-induced vascular remodeling and decreases vascular stabilization [17, 22]. Furthermore, Ang-2 inhibits vascular integrity by competitively inhibiting Ang-1–induced Tie2 activation [23, 24]. Additional lines of evidence suggest that Ang-2 supports angiogenesis by binding the Tie2 receptor in monocytes [25]. In addition, increasing evidence suggests that Ang-2 promotes recruitment of intratumoral macrophages and promotes PD-L1 expression in M2-polarized macrophages [26].

The intertwined regulation of neoangiogenesis and the immune system can offer therapeutic opportunities, and this is of particular interest since checkpoint inhibitors are incorporated into the treatment of HCC, a highly vascular malignancy [27]. Ang-1 and Ang-2 show promise as prognostic markers for HCC, and a study of plasma biomarkers from the SHARP trial identified circulating Ang-2 levels as the only independent predictor of survival in HCC patients treated with sorafenib or placebo [14, 24]. Similarly, our group reported the predictive significance of Ang-2 in a clinical trial of bevacizumab and erlotinib combination in advanced HCC [28].

The goal of the current study was to determine the prognostic value of plasma levels of Ang-1 and Ang-2 in HCC and to explore the diagnostic utility of plasma Ang-1 and Ang-2 in differentiating cirrhosis from HCC. We examined the prognostic roles of plasma Ang-1 and Ang-2 levels by comparing OS of patients with low versus high plasma Ang-1 and Ang-2 levels to determine whether plasma Ang-1 and Ang-2 were associated with clinicopathologic characteristics.

## RESULTS

**Figure 1:**
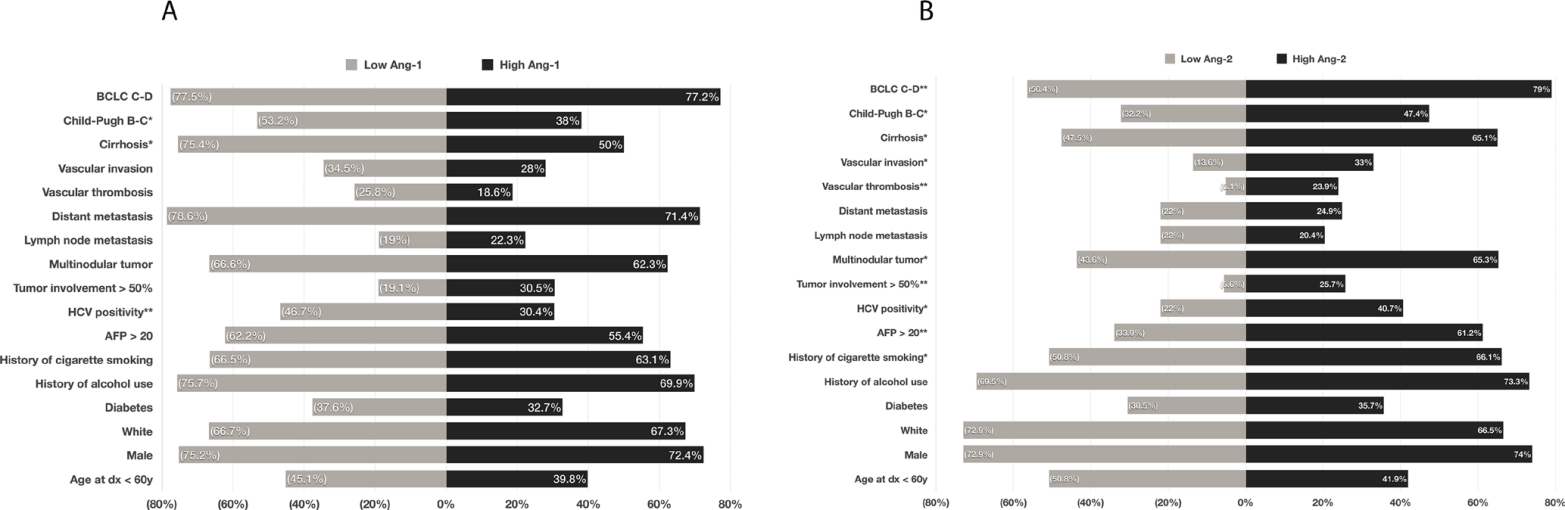
Comparison of prevalence of risk factors, epidemiological parameters, demographic characteristics, and clinicopathological parameters between patients with a low plasma levels of Ang-1 and those with Ang-1 (**A**); and between those with low plasma levels of Ang-2 and those with high Ang-2 (**B**). ^*^p < .05, ^**^p < .001.

Table 1 summarizes the baseline clinical and pathologic characteristics of the 767 HCC patients included in our analysis. Most patients in the study population were older than 60 years (57.4%), and the male-to-female ratio was 2.8:1. Hepatitis in 65.2% and alcohol consumption in 73.0% of patients were the most prevalent risk factors for HCC. Of the 767 HCC patients, 189 (24.6%) had distant metastasis at initial presentation. Most (76.6%) had stage C-D of the Barcelona Clinic Liver Cancer (BCLC) HCC classification systems. Comparison between low (*n* = 415 patients) and high (*n* = 352 patients) plasma Ang-1 levels showed statistically significant differences between the levels based on the presence of cirrhosis, Child-Pugh score, and hepatitis C virus (HCV) positivity (Figure 1A). Comparison between low (*n* = 59 patients) and high (*n* = 708 patients) plasma Ang-2 levels demonstrated statistically significant differences between the levels based on the presence of cirrhosis, Child-Pugh score, the presence of vascular invasion and vascular thrombosis, tumor involvement >50% of the liver, multinodularity, alpha-fetoprotein (AFP) levels, HCV positivity, and history of cigarette smoking (Figure 1B).

**Table 1:** Demographic characteristics, risk factors, and clinicopathological characteristics of 767 HCC patients

Variables	HCC patients (*n* = 767)	
	No. of patients	%
**Age at diagnosis (years)**		
**≤60**	327	42.6
**>60**	440	57.4
**Gender**		
**Male**	567	73.9
**Female**	200	26.1
**Race**		
**White**	514	67.0
**Non-white**	253	33.0
**Hepatitis status**		
**HCV only**	301	39.2
**HBV only**	88	11.5
**HCV and HBV**	111	14.5
**History of cigarette smoking**	498	64.9
**History of alcohol consumption**	560	73.0
**History of diabetes**	271	35.3
**AFP level ≥ 20 ng/dl**	453	59.1
**AFP level ≥ 400 ng/dl**	251	32.7
**Presence of vascular invasion**	241	31
**Presence of vascular thrombosis**	172	22.4
> 50% tumor involvement^*^	180	23.5
**Distant metastasis**	189	24.6
**Lymph node metastasis**	157	20.4
**Adjacent organ invasion**	27	3.5
Multi-nodularity^*^	474	61.8
**Tumor differentiation**		
**Well-differentiated**	193	25.2
**Moderately differentiated**	211	27.5
**Poorly differentiated**	120	13.0
**Fibrolamellar**	13	1.6
**Clear Cell**	7	0.9
**Presence of cirrhosis**	489	63.7
**Child-Pugh class**		
**A**	412	53.7
**B**	299	39.0
**C**	56	7.3
CLIP staging^*^		
**Stage 0–2**	485	63.2
**Stage 3–6**	282	36.8
**BCLC staging**		
**Stage 0-B**	172	22.4
**Stage C-D**	588	76.6
TNM staging^*^		
**Stage I-II**	253	33.0
**Stage IIIA-IIIB**	225	29.3
**Stage IIIC-IVB**	266	34.7
**Frontline treatment**		
**Surgery or transplant**	129	16.8
**Local therapy**	258	33.6
**Systemic therapy**	319	41.6
**Best supportive care**	60	7.8

**Figure 2:**
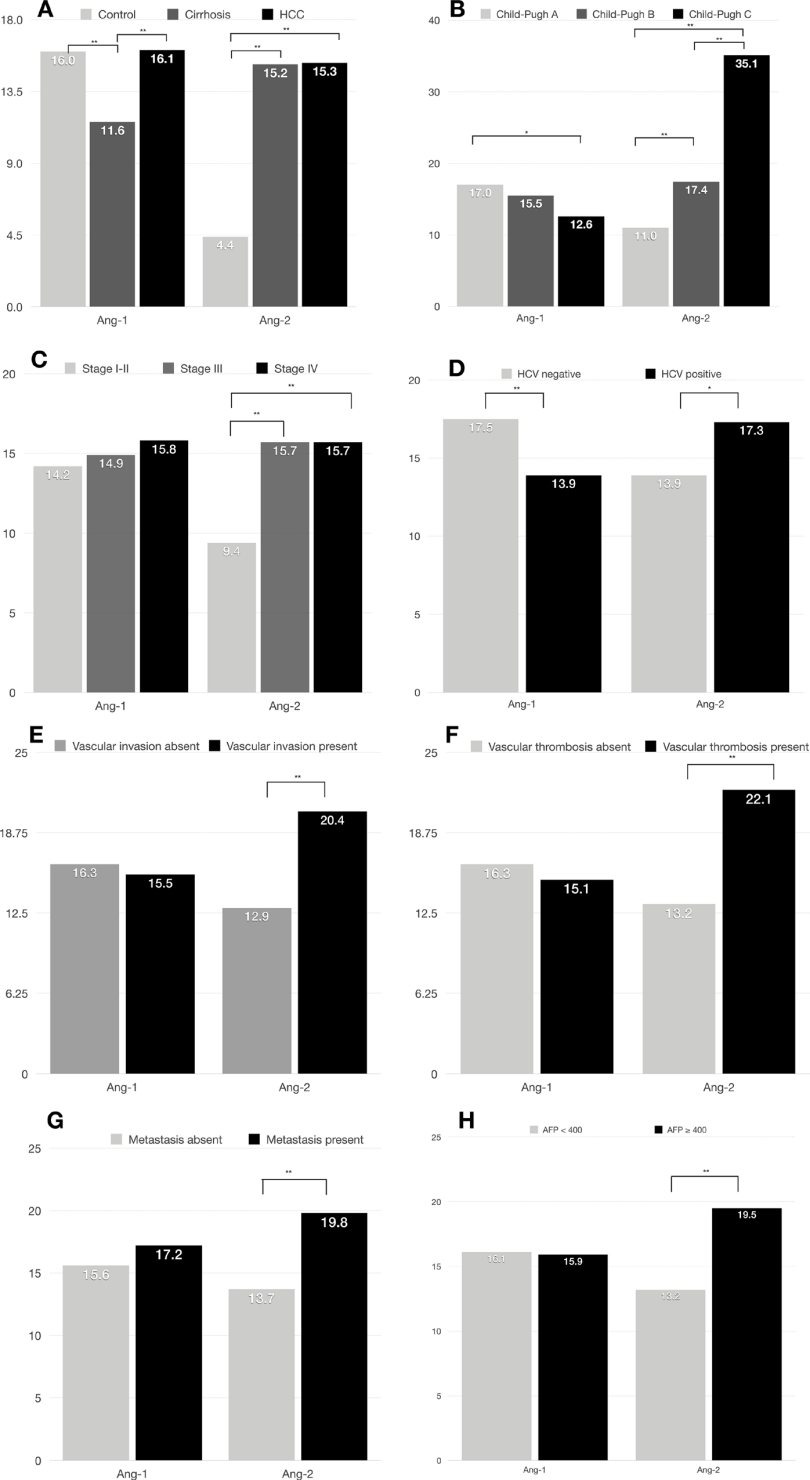
Median plasma Ang-1 and Ang-2 levels by clinical characteristics in 767 HCC patients (**A**) Ang-1 and Ang-2 levels correlate with HCC and cirrhosis; (**B**) Ang-1 and Ang-2 levels correlate with Child-Pugh score; (**C**) Ang-2 levels correlate with TNM staging; (**D**) Ang-1 and Ang-2 levels correlate with HCV positivity; (**E**) Ang-2 levels correlate with vascular invasion; (**F**) Ang-2 levels correlate with presence of vascular thrombosis; (**G**) Ang-2 levels correlate with presence of distant metastasis; (**H**) Ang-2 levels correlate with higher AFP levels. ^*^p < .05, ^**^p < .001.

Plasma Ang-1 levels were significantly lower in cirrhotic patients (mean = 11.6 ng/ml; 95% CI, 10.2–13.0 ng/ml) than in healthy controls (mean = 16.0 ng/ml; 95% CI, 14.9–17.1 ng/ml) and in HCC patients (mean = 16.1 ng/ml; 95% CI, 15.4–16.7 ng/ml) (*p* < 0.001) (Figure 2A). In contrast, plasma Ang-2 levels were significantly lower in healthy controls (mean = 4.4 ng/ml; 95% CI, 4.2–4.7 ng/ml) than in cirrhotic (mean = 15.2 ng/ml; 95% CI, 12.6–17.8 ng/ml) and in HCC patients (mean = 15.3 ng/ml; 95% CI, 14.1–16.4 ng/ml) (*p* < 0.001) (Figure 2A). In addition, mean plasma Ang-2 levels were significantly more elevated in HCC patients with a higher Child-Pugh score, higher TNM-stage, HCV positivity, presence of vascular invasion and thrombosis, presence of metastasis, and higher AFP levels, whereas plasma Ang-1 was significantly lower in patients with a more advanced Child-Pugh score and TNM staging (Figure 2A–2H).

**Figure 3:**
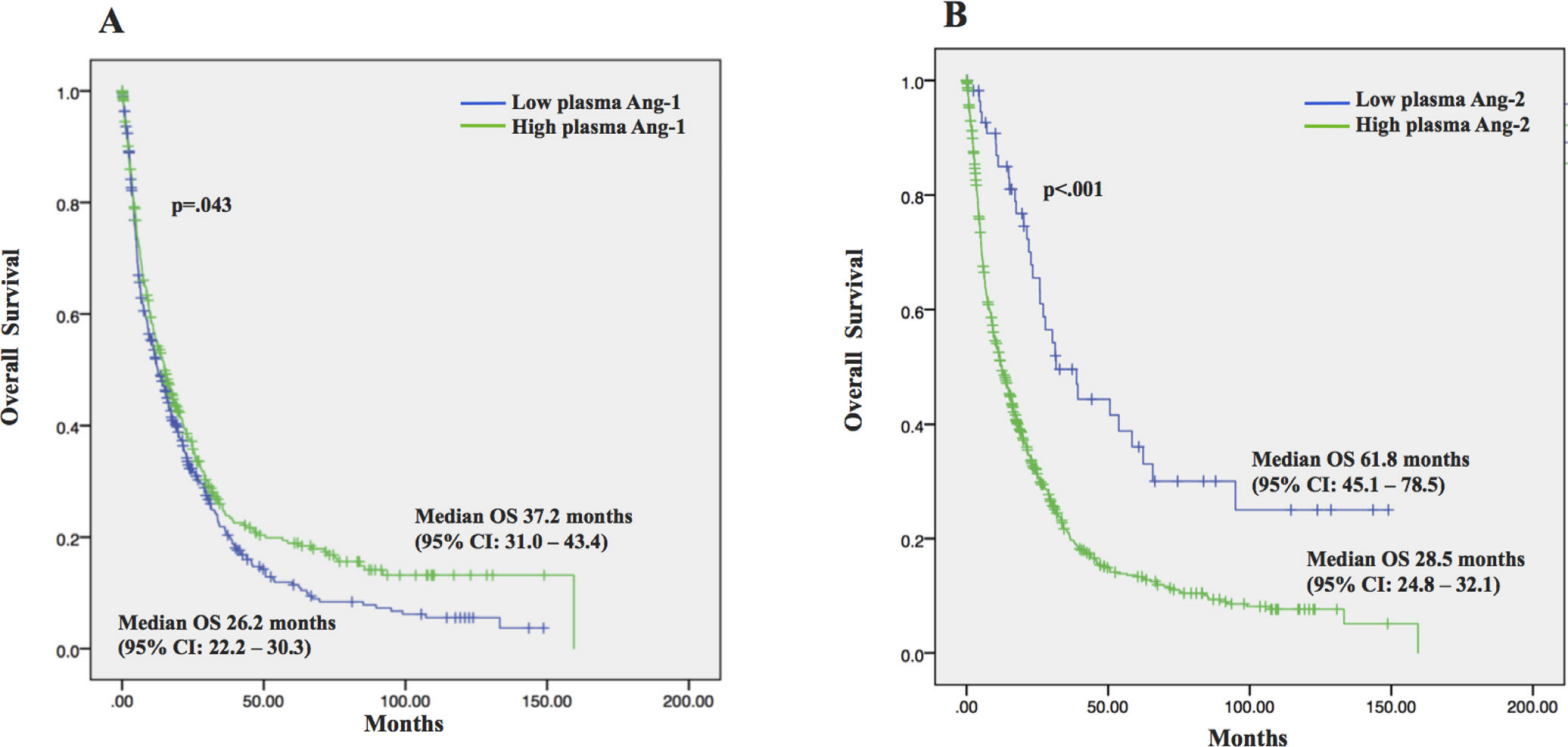
Overall survival (OS) and the 95% confidence interval (CI) in 767 HCC patients (**A**) Patients with high plasma Ang-1 had significantly longer OS as compared to those with low plasma Ang-1; (**B**) Patients with low plasma Ang-2 had significantly longer OS as compared to those with low plasma Ang-2.

Finally, our results demonstrated that plasma Ang-1 and Ang-2 levels correlated with OS. Patients with higher plasma Ang-1 levels had significantly better OS than those with low plasma Ang-1 levels. Median OS was 37.2 months (95% CI, 31.0–43.4 months) for those with high Ang-1 compared with 26.2 months (95% CI, 22.2–30.3 months) for those with low levels (*p* = 0.043) (Figure 3A). Antagonistically, lower plasma Ang-2 levels correlated with prolonged OS. Median OS was 61.8 months (95% CI, 45.1–78.5 months) for those with low plasma Ang-2 compared with 28.5 months (95% CI, 24.8–32.1 months) for those with high levels (*p* < 0.001) (Figure 3B).

## DISCUSSION

To the best of our knowledge, we herein describe the largest cohort correlating plasma levels of Ang-1 and Ang-2 with clinical and prognostic characteristics of HCC. Our current study indicates that higher plasma Ang-2 and lower plasma Ang-1 levels are risk factors for shorter OS in HCC. Consistent with our findings, data from clinical trials including our phase II study with bevacizumab and erlotinib [28] in addition to an analysis of plasma biomarkers of patients enrolled in the phase III randomized SHARP trial demonstrated that elevated serum Ang-2 was an independent risk factor for shorter OS in both the sorafenib and the placebo arms [14].

Our results also showed that plasma levels of Ang-1 was significantly lower in cirrhotic patients than in HCC patients and in healthy controls, whereas plasma Ang-2 was significantly more elevated in cirrhotic and HCC patients than in healthy controls. There were no significant differences in Ang-2 levels in cirrhotic vs HCC patients. These findings suggest that Ang-1 and Ang-2 can jointly be explored as noninvasive diagnostic biomarkers of HCC and liver disease. Consistent with our results, a retrospective study by Hernández-Bartolomé *et al*. [29] analyzing 179 patients had previously demonstrated that although Ang-1 was decreased in cirrhotic compared with non-cirrhotic patients, Ang-2 was significantly increased as the stage of liver disease progressed. Moreover, these authors demonstrated that the ratio of serum Ang-2 to Ang-1 displayed notable accuracy (82.1%) for the diagnosis of cirrhosis at the optimal cut-off. In contrast to our results, Scholz *et al*. demonstrated that Ang-2 levels were significantly higher in HCC patients than in cirrhotic patients [30].

Our study demonstrated that higher plasma Ang-2 levels were associated with advanced clinicopathologic features, including higher TNM stage, higher Child-Pugh score, and higher chance of metastasis. In agreement with our findings, the above-mentioned biomarker analysis of the SHARP trial identified correlation between circulating Ang-2 levels and the presence of macrovascular invasion and higher AFP levels [14]. Also consistent with our findings, Dias-Sanchez *et al*. [31] demonstrated that serum Ang-2 levels in 32 HCC patients correlated with vascular invasion and thrombosis, AFP levels, and advanced BCLC staging.

Two additional interesting elements should be considered with regard to the role of Ang-2 as a noninvasive biomarker for HCC. First, therapies blocking Ang-2 are currently under development (Table 2) [32–45]. Second, the role of Ang-2 as a noninvasive biomarker in HCC might expand with incorporation of immunotherapy in the therapeutic arsenal for HCC [13]. Previous studies have suggested that Ang-2 may contribute to resistance mechanisms to immune checkpoint therapy by enhancing tumor recruitment of monocytes and macrophages and by upregulating PD-L1 expression in tumor-associated macrophages [26]. Recent data also demonstrated that concurrent neutralization of VEGFA and Ang-2 promotes the development and deployment of antitumor immunity in mouse models of cancer, expanding the rationale for trials combining Ang-2–directed therapy with checkpoint inhibitors [46]. This is very relevant to the current paradigm shift in systemic therapy options for HCC which has led to approval of nivolamb recently in HCC.

**Table 2:** Ang-2-targeted therapies in clinical development for cancer therapy (available at http://www.clinicaltrials.gov, accessed July 2018)

Drug name	Description	List of clinical trials	Status
MEDI3617	Fully humanized IgG1k mAb that binds to human Ang-2	Phase I Trial of Tremelimumab Plus MEDI3617 in Patients With Unresectable Stage III / IV Melanoma	Active, not recruiting
		Phase 1/1b, Open-Label, Dose-Escalation and Expansion Study to Evaluate the Safety and Antitumor Activity of MED3617 as Single-Agent or in Combination in Adult Subjects With Advanced Solid Tumors	Completed, with results [25]
Trebananib (AMG386)	Fc-fusion peptibody that prevents Tie2 receptor activation through binding of both Ang-1 and Ang-2	Phase 2 study of trebananib with and without bevacizumab for patients with recurrent glioblastoma	Completed, with results [26]
		Phase I Trial Study of Trebananib in Relapsed Solid Tumors, Including Primary Tumors of the Central Nervous System ADVL1115: A Children's Oncology Group Phase I Consortium Report	Completed, with results [27]
		Phase II Study of First-Line Trebananib Plus Sorafenib in Patients with Advanced Hepatocellular Carcinoma	Completed, with results [28]
		ENGOT-ov-6/TRINOVA-2: Randomised, double-blind, phase 3 study of pegylated liposomal doxorubicin plus trebananib or placebo in women with recurrent partially platinum-sensitive or resistant ovarian cancer	Completed, with results [29]
		Final results of a phase 3 study of trebananib plus weekly paclitaxel in recurrent ovarian cancer (TRINOVA-1): Long-term survival, impact of ascites, and progression-free survival-2	Completed, with results [30]
		Trebananib (AMG 386) in Combination With Sunitinib in Patients With Metastatic Renal Cell Cancer: An Open-Label, Multicenter, Phase II Study	Completed, with results [31]
		Phase II trial of Trebananib (AMG 386) in patients with persistent/recurrent carcinoma of the endometrium	Completed, with results [32]
		Pharmacokinetic drug-drug interaction study of the angiopoietin-1/angiopoietin-2-inhibiting peptibody trebananib (AMG 386) and paclitaxel in patients with advanced solid tumors	Completed, with results [33]
		Trebananib (AMG 386) plus weekly paclitaxel with or without bevacizumab as first-line therapy for HER2-negative locally recurrent or metastatic breast cancer: A phase 2 randomized study	Completed, with results [34]
		A phase 1b, open-label study of trebananib plus bevacizumab or motesanib in patients with solid tumours.	Completed, with results [35]
		Phase II study of the angiopoietin 1 and 2 peptibody trebananib for the treatment of angiosarcoma.	Completed, with results [36]
		A Phase lb Study of the Safety, Feasibility, and Pharmacokinetics of AMG386 Alone and in Combination With Low Dose Cytarabine in Acute Myeloid Leukemia (AML) Patients	Completed, no results
		Phase Ib Study to Test the Safety and Potential Synergy of Pembrolizumab (Anti-PD-1) and AMG386(Angiopoietin-2 (Ang-2) in Patients With Advanced Solid Tumor	Active, recruiting
		I-SPY 2 TRIAL: Neoadjuvant and Personalized Adaptive Novel Agents to Treat Breast Cancer (I-SPY 2)	Active, recruiting
Nesvacumab (REGN910)	Fully human IgG1 mAb that selectively binds Ang-2 with high affinity (24 pmol), but does not bind to Ang-1	A Phase I First-in-Human Study of Nesvacumab (REGN910), a Fully Human Anti-Angiopoietin-2 (Ang2) Monoclonal Antibody, in Patients with Advanced Solid Tumors.	Completed, with results [37]
		A Phase 1b Study of Combined Angiogenesis Inhibition by Administering REGN910 and Aflibercept (Ziv-aflibercept) in Patients With Advanced Solid Malignancies	Completed, no results
Vanucizumab (RG7221)	Bispecific mAb targeting VEGF-A and Ang-2	First-in-Human Phase I Study of Single-agent Vanucizumabin Adult Patients with Advanced Solid Tumors	Completed, with results [38]
		An Open-label, Multi-center, Dose Escalation Phase I Study of Single Agent RO5520985 (Vanucizumab), and in Combination With Atezolizumab, Administered as an Intravenous Infusion in Patients With Locally Advanced or Metastatic Solid Tumors	Completed, no results
		A Phase II, Multicenter, Randomized, Double-Blind Study to Evaluate the Efficacy and Safety of RO5520985 (Vanucizumab) Plus FOLFOX Versus Bevacizumab Plus FOLFOX in Patients With Previously Untreated Metastatic Colorectal Cancer	Completed, no results
		An Open-Label, Multicenter, Dose Escalation Phase Ib Study With Expansion Cohorts to Evaluate the Safety, Pharmacokinetics, Pharmacodynamics, and Therapeutic Activity of RO7009789 in Combination With Vanucizumab in Patients With Metastatic Solid Tumors	Active, recruiting
LY3127804	Humanized lgG4 isotype mAB that selectively binds to Ang-2	A Phase 1 Study of LY3127804 as Monotherapy and in Combination With Ramucirumab in Patients With Advanced Solid Tumors	Active, not recruiting

To the best of our knowledge, our current study provides the first report of the correlation of plasma Ang-1 levels with clinicopathologic and prognostic features in HCC. Ang-1 expression quantified by mRNA was shown to correlate with tumor vascularity in previous reports [24]. In addition, circulating levels of Ang-1 have demonstrated prognostic correlation in other cancers, such as cervical, peritoneal mesothelioma, and nasopharynx and larynx carcinomas [47–49].

Therefore, taken together, our results indicate that circulating Ang-1 and Ang-2 levels correlate both with the presence of cirrhosis and with advanced HCC clinicopathologic features, suggesting also a role in tumor progression. These findings complement results from previous studies. First, previous data has demonstrated that Ang-1 secreted by hepatic stellate cells contributes to liver fibrosis, and circulating Ang-1 and Ang-2 levels have been described as non-invasive biomarkers of cirrhosis in patients with chronic hepatitis C infection, highlighting the close relationship between angiogenesis and liver fibrosis [31, 50]. Second, targeting Tie-2, the receptor for Ang-1 and Ang-2, promote microvascular stability and decrease angiogenesis, and a selective Ang-2 inhibitor has been shown to reduce vascular growth by 46% and tumor size by 62% over a period of 26 days in preclinical models, demonstrating the role of this signaling pathway in HCC progression and further establishing angiopoietin as a potential target for therapy development (Table 2) [51].

In summary, we have demonstrated that circulating levels of the proangiogenic cytokines Ang-1 and Ang-2 could be promising noninvasive prognostic and diagnostic markers in HCC. Independent validation studies with serial plasma measurements are needed to further characterize the significance of plasma angiopoietins levels, not only as prognostic markers, but even more critical, as predictors of response or resistance to therapies, particularly in the era of immunotherapy and antiangiogenesis-directed HCC therapy.

## MATERIALS AND METHODS

### Patients and specimens

The current analysis is part of a case-control, hospital-based study approved by the Institutional Review Board (IRB) of The University of Texas MD Anderson Cancer Center. We obtained written informed consent from all study subjects. With IRB approval, we prospectively collected clinicopathologic data and plasma samples from 767 patients treated at MD Anderson Cancer Center between 2001 and 2014. All patients were treatment-naïve. We have recorded the following characteristics from patients’ medical records, at the time of blood collection: demographic parameters, HCC risk factors (such as cirrhosis, alcohol consumption, and hepatitis B or C infection), HCC treatment modalities received, survival data, and tumor variables such as number of liver nodules, size of each individual tumor, tumor grade and differentiation, the presence of macrovascular invasion, extrahepatic metastasis, and the percentage of tumor-occupied liver area. Information regarding disease staging was also collected with use of three recognized HCC classification systems: (1) the Barcelona Clinic Liver Cancer (BCLC), (2) the Cancer of the Liver Italian Program (CLIP), and (3) the AJCC tumor-node-metastasis (TNM) system.

There were two accepted means for the diagnosis of HCC: by biopsy and pathologic examination or by typical characteristics on contrast-enhanced cross-sectional imaging in cirrhotic patients (computed tomography or magnetic resonance imaging). Patients were included regardless of comorbid conditions. Cirrhotic patient controls consisted of 75 patients who were selected to represent patients in various stages of cirrhosis and Child-Pugh scores, with multiple causes of underlying liver damage. Controls were healthy volunteers (*n* = 200) with no apparent laboratory or imaging evidence of liver damage.

### Measurement of plasma Ang-1 and Ang-2

Ang-1 and Ang-2 plasma levels were measured in plasma samples with use of a commercially available multiplex immunoassay (Myriad, Human Discovery MAP, v3.3, Austin, TX, USA). All samples were collected prior to first cancer-directed treatment. There was no collection of samples after treatment, and no patient had a second measurement of circulation angiopoietin level at another time-point.

### Statistical analysis

Statistical analyses were performed with use of Stata software (Stata Corp, College Station, TX, USA), and univariate analyses for categorical variables were performed by using the X^2^ or Fisher exact test. An analysis of variance (ANOVA) comparison test was utilized to determine differences in serum biomarker levels between groups. The cutoff value for low versus high Ang-1 and Ang-2 levels was set at the median levels of each angiogenic factor in the control group. The Kaplan–Meier method was used to estimate OS and survival results were compared by using the log-rank test. We considered *p* values of less than 0.05 as statistically significant.

## Abbreviations

Ang-1: angiopoietin-1
Ang-2: angiopoietin-2
HCC: hepatocellular carcinoma
mAB: monoclonal antibody
OS: overall survival
